# Time to re-think our strategy with musculoskeletal disorders and workstation ergonomics

**DOI:** 10.4102/sajp.v77i1.1490

**Published:** 2021-01-14

**Authors:** Vanessa S. Redivo, Benita Olivier

**Affiliations:** 1 Department of Physiotherapy, Faculty of Health Sciences, University of the Witwatersrand, Johannesburg, South Africa

**Keywords:** musculoskeletal disorders, ergonomics, visual-display-unit users, ROSA, psychosocial factors, effort-reward imbalance

## Abstract

**Background:**

The dramatic increase in visual display units (VDU) in the workplace over a 20-year period is linked to the increased prevalence of musculoskeletal disorders (MSDs).

**Objectives:**

The objective of our study was to compare ergonomic risk factors and work-related psychosocial factors in VDU users with and without MSD.

**Methods:**

Participants, with and without MSD, working with VDU for more than 4 h a day completed the Nordic Musculoskeletal Questionnaire and the Effort-Reward Imbalance Model and Over-commitment Questionnaire. The workstation of each participant was assessed for ergonomic risk factors using the Rapid Office Strain Assessment (ROSA).

**Results:**

Sixty-eight VDU users with and 68 without MSDs participated. The workstation ergonomic risk factors as measured with the ROSA were similar for the two groups: 4.5 ± 1.0 for the MSD group and 4.3 ± 0.8 for the reference group (*p* = 0.10). The work-related psychosocial factors, namely over-commitment, were higher in the MSD group (14.9 ± 3.1) than in the reference group (13.8 ± 3.4; *p* = 0.041).

**Conclusions:**

As over-commitment is an indication of intrinsic factors and personal characteristics, the significant difference between the MSD group’s over-commitment score and that of the reference group suggests that interventions to empower individuals are needed.

**Clinical implications:**

Physiotherapists should only adjust ergonomic workstation risk factors when established as contributory to MSD, and should be cognisant of work-related or individual psychosocial factors that may impact the patient with MSD. The use of ergonomic advice to patients with MSD should be performed with caution, taking all the work place risk factors for MSD into account.

## Introduction

There has been a dramatic increase in visual display units (VDU) in the work place over a 20-year period, as much as doubling the percentage of users (Kaliniene et al. 2013; Sonne, Villalta & Andrews 2012; Wahlstrom 2005). A VDU user is defined as an individual working with a VDU that involves the use of a keyboard and mouse, or both (Collins Dictionary 2018). The prevalence of musculoskeletal disorders (MSDs) in VDU users has increased; one of the many contributory factors includes the increased use of VDU (Ranasinghe et al. 2011). This increase in MSD has resulted in an increase in sick days (absence from work), reduced efficiency, an increased burden of disease and loss to the economy (Green 2008; Matos & Arezes 2015; Van Eerd et al. 2016). Work-related MSD continues to pose challenges to the health system (Silva et al. 2014) and leads to disability and compensation claims (Maakip, Keegel & Oakman 2017; Widanarko et al. 2014).

The reported prevalence rate of MSD may also vary according to the type of occupation and the sample population studied (De Cássia Pereira Fernandes et al. 2016; Huisstede et al. 2006). Visual display unit users have shown increased upper quadrant prevalence rates in comparison to lower back pain (Ardahan & Simsek 2016; Das & Ghosh 2010; Green 2008; Ranasinghe et al. 2011; Wu et al. 2012), specifically, disorders of the neck (Green 2008; Wu et al. 2012). Multi-site pain is also prevalent in VDU users (Neupane & Nygård 2017; Oha et al. 2014).

Work-related MSD has been documented to be multi-factorial (Matos & Arezes 2015; Oha et al. 2014; Sonne et al. 2012; Wahlstedt et al. 2010), and includes physical factors like muscular load, non-neutral postures, static postures, extreme positions, repetitive movements, force, visual demands, the duration of time spent at work, the duration of time spent in front of the VDU, the workstation set-up, as well as associated psychosocial work demands, such as mental stress, job control, support, work style and technique, perceived tiredness, inactivity or individual factors such as smoking, socioeconomic factors, sex and personal demographics (Bruno Garza & Young 2015; Das & Ghosh 2010; Green 2008; Korhonen et al. 2003; Ranasinghe et al. 2011; Rodrigues, Leite, Lelisa & Chaves 2017; Sun, Nimbarte & Motabar 2017; Wahlstedt et al. 2010; Wahlstrom 2005; Wu et al. 2012; Zakerian & Subramanian 2011).

A poor ergonomic workstation has been shown to increase muscular load and muscular activity causing an increase in MSD (Sun et al. 2017; Wahlstrom 2005). The physical factors or load on the biomechanical system are hypothesised to cause tissue damage and inflammation, thus resulting in MSD (Bruno Garza & Young 2015). Quantifying the ergonomic risk of a workstation is a method to cost-effectively implement and manage MSD in the work place (Sonne et al. 2012) and a plausible deduction would seemingly be to address the ergonomic workstation set up as a method to prevent, as well as address MSD (Ranasinghe et al. 2011). However, there is still conflicting evidence that shows no correlation between workstation set up and MSD (Coelho et al. 2015; Klussmann et al. 2008; Lima & Coelho 2018; Wu et al. 2012). Research that supports the general ergonomic advice provided to patients in South Africa is lacking, thus warranting further investigation in the South African population.

With regard to the workstation set up amongst VDU users, the comprehensive review, conducted by Woo, White and Lai (2016), included ergonomic guidelines and workstation arrangements from Australia, Canada, United States, Europe and Hong Kong. Woo et al. (2016) note that the studies use a baseline of anthropometrics and biomechanics to establish guidelines for computer workstation design. However, with the differences in anthropometrics in each country, the guidelines should allow optimal adjustment and be flexible for each end user. The review by Woo et al. (2016) does not cover any African, let alone South African standards, guidelines or studies. There is a need, therefore, to research the South African population anthropometrics within the workstation design framework in order to establish South African guidelines.

Specific ergonomic guidelines, including a neutral posture, monitor position, appropriate work surface, the use of supportive accessories, the correct type of chair and a satisfactory sitting position have been suggested in order to minimise the physical risk factors for MSD in the VDU user (Woo et al. 2016). The correlation of neck pain, disability and forward head posture has been established (Kim & Kim 2016), and could be indicative of the effect of non-neutral postures. A constant neck flexion angle with the neck being held in a bent position has also been determined as a risk factor amongst VDU users for MSD of the neck (Sun et al. 2017; Wu et al. 2012). A neck flexion angle of 45° has been found to significantly affect the upper and lower back, shoulders and feet and may lead to pain (Celik et al. 2018). The use of laptops increases neck flexion angles and limits the adjustability of the workstation (Werth & Babski-Reeves 2014), and could affect work postures, bringing about discomfort and MSD. Sitting is a risk factor for lower back pain and other MSDs (Celik et al. 2018; Green 2008; Silva et al. 2014) and sitting for long periods in addition increases the risk for lower back pain (Silva et al. 2014).

Psychosocial factors influencing this multi-factorial problem have been an area of increased interest. The literature assessing psychosocial risk factors associated with MSD in VDU users highlights the following: computer-related problems, employees’ level of job control and social interaction (Zakerian & Subramanian 2011), increased work demand, greater work experience (Kaliniene et al. 2013), considerable demands associated with the job, limited job influence and development, low level of job satisfaction, poor interpersonal relationships and leadership (Yue et al. 2014), increased mental demands, low job control (Devereux, Vlachonikolis & Buckle 2002), limited social support (Devereux et al. 2002; Wahlstedt et al. 2010), high demands and stress (Wahlstedt et al. 2010).

A tool to measure work-related psychosocial risk factors includes the Effort-Reward Imbalance Model and Over-commitment. The questionnaire was designed to clarify the impact of social and psychosocial factors on health and disease (Siegrist 1996), and has been successfully used on a mixed sample group, including healthcare workers, random sample of the general population, office workers, call centre operators, blue collar workers and police officers (De Jonge et al. 2008; Koch et al. 2014; Landolt et al. 2017). An Effort-Reward Ratio is used to assess potential imbalances between high effort and limited rewards (Siegrist 2012). Reward is the reciprocation of such effort, and has three defined sub-scales, namely esteem, promotion and security (Siegrist, Li & Montano 2014). Over-commitment, an intrinsic factor, is said to refer to individuals, who have an excessive work-related commitment, as well as a need for approval (Siegrist et al. 2004) and could be elevated in those who have a misconception of perceived demands, as well as poor personal coping strategies (Siegrist 2012). Over-commitment can be defined as a person-specific component (Van Vegchel et al. 2005), where Effort-Reward is related to structural work components (Siegrist et al. 2004).

Discovering which factors play a role in MSD, and the relationship between these factors and MSD could result in better intervention methods to address and manage MSD. The relationship between MSD, ergonomic workstation set up and psychosocial factors has not been established in the VDU user in the work place in South Africa. To the authors’ knowledge, there has been only one study conducted in South Africa within a VDU population and ergonomic set-up. This was a case study, and pain and discomfort were found to decrease with an optimal ergonomic workstation set-up (Van Vledder & Louw 2015). Other studies related to MSD in the South African population are often conducted amongst blue-collared workers. When comparing high income and middle-to-low income countries, there appear to be differences between prevalence rates and the site or location and the type of MSD observed in public sector office workers. The reason for these differences in MSD in the office worker may be because of specific sociocultural factors (Maakip et al. 2017). This warrants further research in the South African population.

The workstation set-up has been mentioned as a risk factor; however, findings in this respect are shown to be inconsistent (Coelho et al. 2015; Klussmann et al. 2008; Lima & Coelho 2018; Wu et al. 2012). As such with the added interest of the influence of psychosocial risk factors, the relationship between ergonomic risk factors and psychosocial risk factors in MSD and a reference group needs to be established. Thus, the objective of our study was to compare ergonomic risk factors and work-related psychosocial factors in VDU users with and without MSD in the South African population.

## Methods

This cross-sectional, observational study was conducted at the workstations of three companies in Pretoria and Johannesburg in the Gauteng province. A sample of convenience consisting of VDU users in the fields of administration, information technology and research was included based on consent received from the human resource departments. The three companies were the only companies that allowed access to the employees of a large group of companies that were initially approached to participate in our study.

Participants were included if they were 18 years old and above, using a VDU at work for more than 4 h a day (Lapointe et al. 2013; Mirzaei et al. 2014; Oha et al. 2014; Poochada & Chaiklieng 2015), working more than 12 months in their current job or a similar occupation. The exposure for 4 h or more was considered to be sufficient exposure to the risk factors for MSD in similar studies of VDU users (Lapointe et al. 2013; Mirzaei et al. 2014; Oha et al. 2014; Poochada & Chaiklieng 2015). Employees who had undergone surgery for MSD in the previous 2 years, or were undergoing mental health treatment, including but not limited to depression and anxiety were excluded. The sample size required to analyse the difference between the MSD and reference group was 128, with 64 participants in each group. This was calculated by G*Power (version 3.1.9.2) with a power probability of 0.8 at the 0.05 alpha level (two-tailed design), using a moderate effect size (Cohen’s *d* = 0.5) and an equal allocation of participants in each group.

## Outcome measures

All participants completed a self-report questionnaire consisting of questions relating to individual demographics (age, sex, height, weight and handedness) using an amended Cultural and Psychosocial Influences on Disability (CUPID) questionnaire (Coggon et al. 2012) as well as the Nordic Musculoskeletal Questionnaire (Kuorinka et al. 1987) to determine MSD. The Nordic Musculoskeletal Questionnaire is also considered to be an adequate screening tool for comparing the sensitivity of the responses to the questionnaire with the results of the clinical examinations. The questionnaire showed a sensitivity to MSD between 66% and 92% (Crawford 2007). The participant was asked whether to his or her knowledge the MSD was related to factors other than work in order to allow for the correct allocation of participants to either the MSD group or the reference group. Should the participant not have recollection of any other cause of MSD injury, it was considered work-related MSD, and only a small number of the participants did not specify the cause of MSD.

Work-related psychosocial factors were assessed using the Effort-Reward Imbalance Model and Over-commitment Questionnaire. High effort and limited rewards produce a ratio of more than 1.0, or non-reciprocity, or limited effort and high level of reward is defined as a ratio less than 1.0. The questionnaire includes 24 questions in total, five questions related to effort, 11 questions related to reward and six for over-commitment on a four-point Likert scale (Siegrist 1996). The Effort-Reward Ratio is calculated by the sum of the efforts as an enumerator and the sum of the rewards as the denominator. The denominator score was multiplied by a correction factor of 0.4545 to adjust for unequal items between effort and reward. Effort, total reward, reward subscales (esteem, promotion and security) and over-commitment were calculated by the sum of the scores of each construct as defined in the questionnaire (Siegrist 2012). Internal consistency in respect of an effort score of alpha 0.74, a reward score of alpha 0.82 and an over-commitment score between 0.73 and 0.78 is considered to be satisfactory (De Jonge et al. 2008)

**FIGURE 1:**
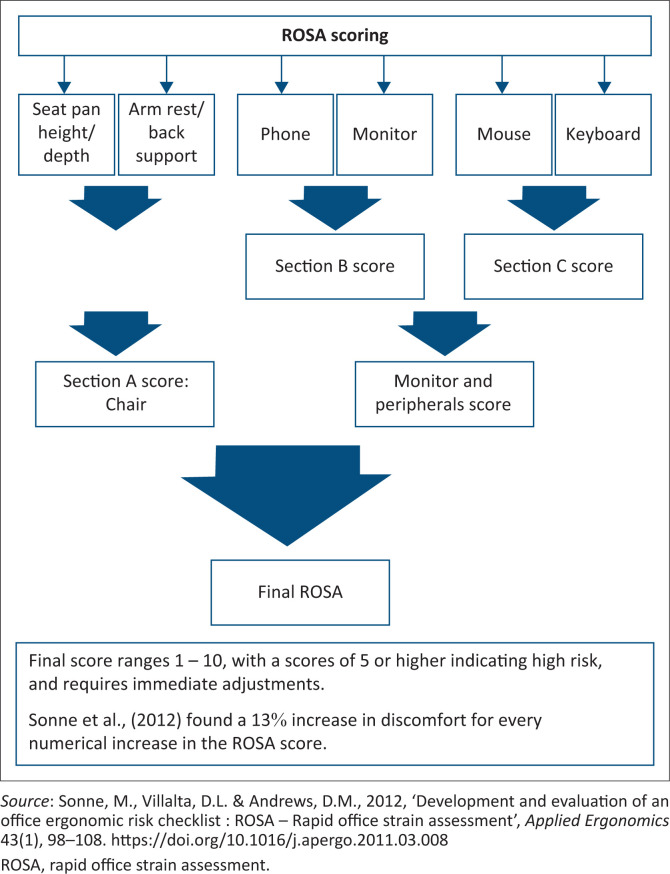
Diagrammatic presentation of the rapid office strain assessment scoring system.

Ergonomic risk factors were measured using the Rapid Office Strain Assessment (ROSA). The ROSA is meant to quickly quantify risks in a typical workstation into low, medium and high. The risk assessment is aimed to assist the user to prioritise changes that are based on ergonomic standards, as well as discomfort scores (Sonne et al. 2012). During the development of the ROSA, the intra and inter-observer reliability levels were assessed, with the intra-observer reliability of the final ROSA score being 0.91 (Sonne et al. 2012). A final score is a calculation of the risk relating to the chair, and peripherals including a score of the monitor, phone, keyboard and mouse. Duration spent using the various components of the workstation affects the overall score. One-hour continuous use or more than 4 h a day results in an additional point awarded to each component. A score of five or higher requires immediate adjustments (Sonne et al. 2012). Considering its specificity to computer use in the work place, ROSA has been used with great success in other studies (Matos & Arezes 2015; Poochada & Chaiklieng 2015). Figure 1 illustrates the ROSA scoring system.

## Procedure

**FIGURE 2:**
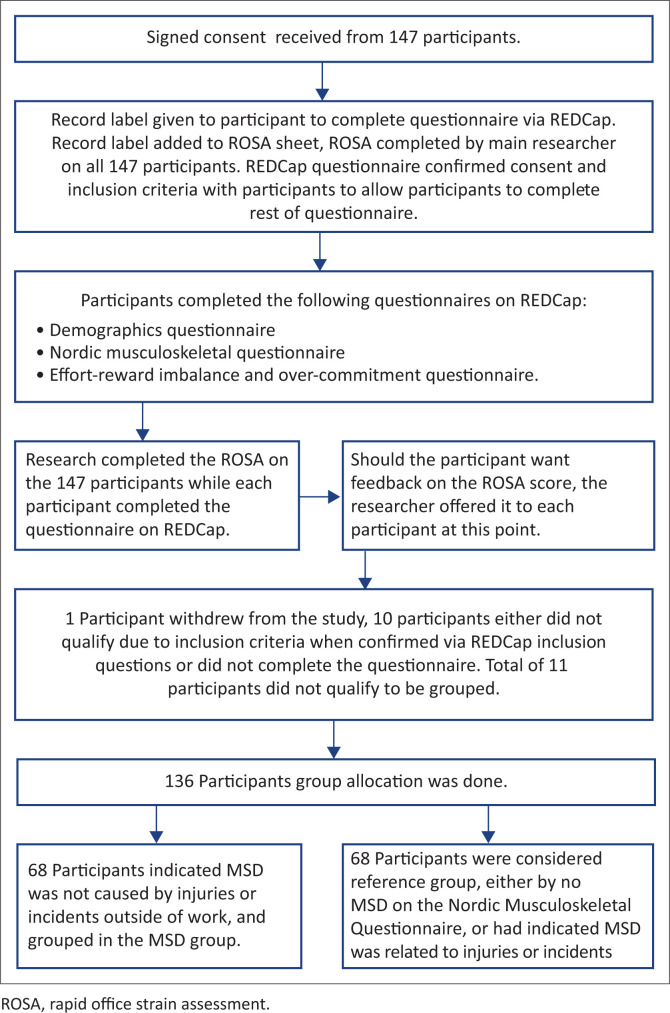
Flow diagram of study procedure.

The study procedure is depicted in Figure 2. Prior to the data collection, permission to access employees was obtained from the human resource departments of the companies in question. A pilot study was performed in order to discern any areas of bias and to streamline the data collection process. As no changes were made to the procedures after the pilot study, data were analysed with the data collected in the main study.

Each participant was included in an onsite informed-consent information session, and only after the participant had signed consent to participate, the link to the online questionnaire was emailed to him or her. Had the employee declined to take part in our study, no link was sent and the first author approached the next employee. Research Electronic Data Capture (REDCap), a data collection and management platform (Harris et al. 2009), was used for online data collection. The participant completed the online self-reported questionnaires at his or her workstation.

Whilst the participants were completing the questionnaire, the first author observed the respective workstations and in each case completed the ROSA (Sonne et al. 2012). The ROSA does not need formal training; however, the first author was proficient in completing the ROSA. The ROSA was presented to all participants, regardless of any MSD complaints. At that point, the first author was blinded to the presence of MSD. Once the participant had completed all questionnaires online, at the end of the session, feedback about ergonomic workstation risk factors and adjustments was given to all willing participants.

Only the first author dealt with the ROSA and ergonomic advice. This allowed for consistency in the application of the assessment tool. The first author completed 147 ROSA assessments; however, 10 participants did not qualify because of the inclusion criteria or did not complete the questionnaire on REDCap, and one participant withdrew from our study without giving a reason. The remaining 136 participants were grouped into the work-related MSD group, or the reference group either with no MSD or non-work related MSD. Two groups of 68 participants were formed.

## Data analysis

Each variable was described in terms of its mean, standard deviation, frequency and percentages, as relevant, in the MSD and the reference group. The variables for individual demographics and anthropometrics included age, height, weight, body mass index (BMI), sex and handedness. Differences between the categorical variables of the MSD group and the reference group were analysed using Chi-squared tests. Independent sample t-tests were run to determine whether there were differences between the mean variable scores for the MSD group and the reference group. Missing data were not replaced and thus ignored in the analyses.

### Ethical consideration

Ethical clearance was sought and obtained from the Human Research Ethics Committee (Medical) of the University of the Witwatersrand, Johannesburg (Clearance Certificate no. M171064).

## Results

### Demographics and anthropometrics

The two groups consisted of 68 participants each (*n* = 136). The MSD group consisted of 31 (45.6%) females, and the reference group consisted of 27 (39.7%) females (*p* = 0.603). The majority of participants were right-handed (MSD group *n* = 62; 91.2%; reference group *n* = 65; 95.6%; *p* = 0.493).

Table 1 reflects the demographics and anthropometrics, where no differences between the two groups in terms of mean values for age, height, weight and BMI and proportions for sex and handedness were found.

**TABLE 1:** Group demographics and anthropometrics (*n* = 136).

MSD group		Reference group		*p*
Demographics	Mean ± SD	Demographics	Mean ± SD	
Age (years) (*n* = 68)	33.3 ± 8.5	Age (years) (*n* = 68)	35.3 ± 8.7	0.167
Height (m) (*n* = 68)	1.7 ± 0.1	Height (m) (*n* = 66)	1.7 ± 0.1	0.992
Weight (kg) (*n* = 68)	75.9 ± 15.7	Weight (kg) (*n* = 67)	75.3 ± 13.6	0.780
BMI (kg/m^2^) (*n* = 68)	26.1 ± 5.3	BMI (kg/m^2^) (*n* = 66)	26.1 ± 4.9	0.986

MSD, musculoskeletal disorders; BMI, body mass index; SD, standard deviation.

### Musculoskeletal disorders

Table 2 shows the prevalence of MSD by location of pain as determined by the Musculoskeletal Nordic Questionnaire. The majority of participants had experienced neck pain in the previous 12 months as well as in the last 7 days.

**TABLE 2:** Previous 12 months and 7 days prevalence in musculoskeletal disorders by location (*n* = 68).

Location	Previous 12 months		Previous 7 days	
	%	*n*	%	*n*
Neck	69.1	47	33.9	23
Shoulder	57.4	39	27.9	19
Upper back	39.7	27	16.2	11
Elbow	5.9	4	2.9	2
Wrists/hands	25	17	8.8	6
Lower back	41.2	28	13.2	9
Knee	19.1	13	7.4	5
Ankle	5.9	4	1.5	1
None	0	0	33.9	23

Participants could indicate more than one site of MSD. More than one site of MSD was classified as multi-site MSD. The MSD group experienced a mean score for multi-site MSD of 2.6 ±1.4. In terms of multi-site pain, 15 participants (22.1%) experienced pain in one site, 22 (32.4%) at two sites, 16 (23.5%) at three sites, six (8.8%) at four sites, seven (10.3%) at five sites, one (1.5%) at six sites and one (1.5%) at seven sites.

### Work-related psychosocial risk factors

Table 3 shows the mean values for scale of effort, total reward, the Effort-Reward Ratio, the reward sub-scales and the over-commitment scores for the MSD group and the reference group, respectively. The Effort-Reward Ratio for the MSD group as well as for the reference group was calculated as less than one; hence there were fewer efforts than rewards. The over-commitment score was significantly different (*p* = 0.041) where the MSD group achieved a higher mean over-commitment score as opposed to the reference group.

**TABLE 3:** Effort-reward imbalance and over-commitment in the musculoskeletal disorders group (*n* = 68) and the reference group (*n* = 68).

Variables	Range	MSD group Mean ± SD	Reference group Mean ± SD	*p*
**Effort-Reward Ratio**	< 1 or > 1	0.9 ± 0.2	0.9 ± 0.2	0.287
**Effort**	6–24	15.6 ± 3.0	14.8 ± 2.3	0.122
**Total reward**	10–40	30.9 ± 3.9	30.8 ± 3.7	0.892
**Reward sub-scales**				
Reward-esteem	4–16	9.6 ± 1.4	9.6 ± 1.3	0.797
Reward-promotion	4–16	11.8 ± 1.9	11.4 ± 1.7	0.587
Reward-security	2–8	6.4 ± 1.0	6.4 ± 1.2	0.877
**Over-commitment**	6–25	14.9 ± 3.1	13.8 ± 3.4	0.041*

MSD, musculoskeletal disorders; SD, standard deviation; Range, minimum to maximum range of scores per construct as defined in the questionnaire.

* , statistically significant.

### Ergonomic risk factors

The mean ROSA scores from both the MSD group and the reference group were under five. The mean final ROSA scores for the MSD group and the reference group were 4.5 ± 1.0 and 4.3 ± 0.8 (*p* = 0.102), respectively. Table 4 shows the number of participants in the MSD group and the reference group in each ROSA final score grouping. Tables 5–7 indicate the sub-scores of the ROSA, Section A, B and C.

**TABLE 4:** Final rapid office strain assessment Score for musculoskeletal disorders group (*n* = 68) and reference group (*n* = 68).

Score	MSD group		Reference group	
	%	*n*	%	*n*
1	20.6	14	23.5	16
2	23.5	16	30.9	21
3	42.7	29	42.7	29
4	10.3	7	2.9	2
5	2.9	2	0	0

MSD, musculoskeletal disorder.

**TABLE 5:** Sub-scores of the rapid office strain assessment (Section A).

Score	Seat pan/height/depth				Arm rest/back support				Section A: Chair			
	MSD		Ref		MSD		Ref		MSD		Ref	
	%	*n*	%	*n*	%	*n*	%	*n*	%	*n*	%	*n*
2	0.0	0	0.0	0	0.0	0	0.0	0	10.3	7	13.2	9
3	69.1	47	72.1	49	17.7	12	16.2	11	29.4	20	30.9	21
4	10.3	17	20.6	14	23.5	16	30.9	21	32.3	22	28.2	26
5	0.0	0	4.4	3	35.3	24	36.8	25	17.7	12	16.2	11
6	1.5	1	2.0	2	17.6	12	16.2	11	7.4	5	1.5	1
7	4.4	3	0.0	0	5.9	4	0.0	0	2.9	2	0.0	0

MSD, musculoskeletal disorders; Ref, reference group.

No comparison between sub-scores was undertaken as only the final ROSA score was validated in the literature. However, the sub-scores are presented to allow for discussion; Zero scores were removed from the table to allow for ease of reading.

Section A: Chair score and peripheral score combined provided the final ROSA score.

**TABLE 6:** Sub-scores of the rapid office strain assessment (Section B).

Score	Monitor				Phone				Section B			
	MSD		Ref		MSD		Ref		MSD		Ref	
	%	*n*	%	*n*	%	*n*	%	*n*	%	*n*	%	*n*
1	0.0	0	0.0	0	100	68	97.1	66	23.5	16	26.5	18
2	10.3	17	27.9	19	0.0	0	1.5	1	32.3	22	35.3	24
3	30.9	21	33.9	23	0.0	0	1.5	1	42.7	29	35.3	24
4	42.7	29	36.8	25	0.0	0	0.0	0	1.5	1	2.9	2
5	1.5	1	0.0	0	0.0	0	0.0	0	0.0	0	0.0	0

MSD, musculoskeletal disorders; Ref, reference group.

No comparison between sub-scores was undertaken as only the final ROSA score was validated in the literature. However, the sub-scores are presented to allow for discussion; Zero scores were removed from the table to allow for ease of reading.

Section B later provided a peripheral score.

**TABLE 7:** Sub-scores of the rapid office strain assessment (Section C).

No.	Mouse				Keyboard				Section C			
	MSD		Ref		MSD		Ref		MSD		Ref	
	%	*n*	%	*n*	%	*n*	%	*n*	%	*n*	%	*n*
2	13.2	9	13.2	9	29.4	20	33.9	23	0.0	0	0.0	0
3	60.3	41	50.0	34	44.1	30	29.4	20	52.9	36	45.6	31
4	26.5	18	36.8	25	22.1	15	33.9	23	11.8	8	20.6	14
5	0.0	0	0.0	0	4.4	3	2.9	2	32.3	22	32.2	22
6	0.0	0	0.0	0	0.0	0	0.0	0	2.9	2	1.5	1

MSD, musculoskeletal disorders; Ref, reference group.

No comparison between sub-scores was undertaken as only the final ROSA score was validated in the literature. However, the sub-scores are presented to allow for discussion; Zero scores were removed from the table to allow for ease of reading.

Section C later provided a peripheral score.

Zero scores were removed from the table to allow for ease of reading. A score of five or more indicates high risk and need for immediate adjustment. Not one participant had a score lower than three, or higher than seven.

## Discussion

No differences in terms of demographics and anthropometrics between the MSD group and the reference group were found. This is in contrast to studies where age has been considered a risk factor (Celik et al. 2018; Collins & O’Sullivan 2015; Kaliniene et al. 2013; Wu et al. 2012). As Kaliniene et al. (2013) noted, there is an increased risk of 2.37 for neck MSD between the ages of 40 and 49 years. The mean age of participants, of whom all had MSD, Collins and O’Sullivan’s (2015) study was also in the early forties, with males and females presenting with this complaint at the ages of 40.1 and 40.3 years, respectively. In our study, both the MSD group and the reference group presented with a young mean age, in the early thirties, and this young mean age could be the attributing factor for our findings.

There was no difference for BMI between the two groups. Both groups were classified as being overweight based on the calculations of the mean BMI score (Khosla & Lowe 1967), with the MSD group and the reference group BMI values at 26.1 ± 5.3 and 26.1 ± 4.9, respectively. An increased BMI is known to be a risk factor for multi-site pain (De Cássia Pereira Fernandes et al. 2016), as well as lumbar pain (Piranveyseh et al. 2016), neck pain (Wu et al. 2012), leg pain in females and wrist pain in males (Celik et al. 2018). Kaliniene et al. (2013) found that an increased BMI is not associated with neck MSD. Although the study by Kaliniene et al. (2013) was a small study sample, the BMI does not seem to be a risk factor for MSD, but the authors felt that BMI could add to the compounding effect in that it constitutes part of the multi-factorial nature of MSD.

Right-handedness in both the MSD and the reference group was determined at 91.2% and 95.6%, respectively. Right-handedness has been linked to MSD (Oha et al. 2014). However, handedness has been regarded as insignificant by Abaraogu et al. (2018). Both of these studies were conducted on VDU populations in a university setting, however the participants demographics were different as Abaraogu et al. (2018) participants were 54.5% woman versus the 85% in the study by Oha et al. (2014). Both the MSD and the reference group in our study were predominantly right-handed, which did not seem to influence MSD in this case, which is similar to the study by Abaraogu et al. (2018). One could speculate that as right-handedness is so prevalent, devices would most likely be set up to be ergonomically sound for right-handed individuals and this could be a contributing factor for the management of associated risks that could lead to MSD. Alternatively, left-handed individuals would have adapted to work within a society that is predominantly right-handed and would, therefore, not be influenced by the handedness factor.

The 12-month prevalence rate is similar to other studies with the highest scores in the neck (69.1%), shoulders (57.4%), lower back (41.2%) and upper back (39.1%), followed by the hands/wrists (25%), knees (19.1%), elbows (5.9%) and ankles/feet (5.9%) (Huisstede et al. 2006; Klussmann et al. 2008; Maakip, Keegel & Oakman 2016; Mirzaei et al. 2014; Oha et al. 2014). The 7-day prevalence rates were found to be slightly higher across all regions, but share the same top four groupings, namely neck (33.9%), shoulders (27.9%), lower back (13.2%) and upper back (16.2%). The difference between the 12-month prevalence and 7-day prevalence could be because of recall. The shared top-four grouping provides an indication of the risk areas for MSD in the VDU user.

Multi-site pain is common in VDU users (Neupane & Nygård 2017; Oha et al. 2014). Our results support these findings as many of those living with MSD indicated that they experience multi-site pain, with 77.9% (*n* = 53) experiencing more than one site of pain. It is a matter of concern that as many as seven participants were found to present with more than five sites of pain, with the majority of participants experiencing two sites of pain. Although the Nordic Musculoskeletal Questionnaire does not establish chronicity, multi-site pain is common in chronic pain individuals (Marchand et al. 2015). Chronic pain could explain this tendency, although chronic pain is complex and further investigation into the mechanisms behind chronic pain is warranted. Alternatively, multi-site pain is said to be a continuum of single-site pain that is sustained by exposure to several risk factors (De Cássia Pereira Fernandes et al. 2016).

The Effort-Reward Ratio in the MSD and the reference group was calculated as less than one, indicating that the rewards are greater than the effort. This is viewed to be positive as it minimised the risk for reduced health, stress and burnout (Koch et al. 2014; Van Vegchel et al. 2005). The effort, reward and reward sub-scores were not different between the two groups.

The over-commitment questions test the ability or inability to withdraw from work and the inclination to provide for an over-the-top effort (Siegrist et al. 2004). Over-commitment is increased in those who have a misconception of the demands of the job, with poor coping strategies (Siegrist 2012) and a distorted perception of their own cost-gains relations (Siegrist 1996). Over-commitment has been associated with poor self-rated health (Siegrist et al. 2004) and intensified stress symptoms (Feldt et al. 2013). The MSD group could be at risk of inflating their own MSD problems through inaccurate recall, and perceived work demands, even though these aspects may be similar for the reference group. Our findings suggest the need to address over-commitment and psychosocial risks in order to maintain an optimal level of occupational health and to minimise disability. Addressing non-reciprocity and over-commitment through psychological detachment, relaxation, mastery and control has been found to be important in the management of the potential risk for burnout and poor recovery (Feldt et al. 2013).

There was no significant difference between the MSD and the reference group’s risk assessment scores. These findings support other studies that also found no association between the workstation set-up and MSD (Coelho et al. 2015; Klussmann et al. 2008; Lima & Coelho 2018; Wu et al. 2012). There are many components to a workstation, and the lack of association between poor ergonomics and MSD may be because of the ROSA assessment method, and components tested. The ROSA has a few challenges which could have contributed to the lack of differentiation between the two groups assessed here. These challenges will be discussed below. The mean value for both groups was a classification of four, thus indicating a medium-risk classification, where scores of five or more require immediate change (Sonne et al. 2012). A score of less than five, however, does not imply that there is no room for improvement; it is still viewed as a risk factor and requires adjustments to the optimum. As only 14 and 16 participants in the MSD group and the reference group, respectively, had scores of three, the remaining participants all had areas to address to minimise risk.

The ROSA also has limitations to the scoring, as one high sub-score can cause a high final score, even though the rest of the workstation is not at risk to the same extent. A score of five or more in the arm and back supports that sub-score assessment would by default result in at least a five final ROSA score. The seat pan height/depth sub-score, a score of six or more on this assessment, would by default result in at least a five final ROSA score.

Not one of the participants had an adjustable seat pan, and hence all would score at least one point in this section. The monitor score reflects the monitor component of the VDU. Risk factors pertain to position, height, distance, non-neutral postures, glare, document holders and duration. All participants were awarded a point for the duration of the work period as, by inclusion, all participants used the VDU for more than 4 h a day. The lowest a participant could score was a value of two – for the correct distance and level at which the monitor was mounted. The distribution of scores is aligned with the use of multiple monitors, a poor desktop set-up and the laptop components to which the participants assessed had access. Most of the participants’ scores increased on account of their unsatisfactory distances from the VDU, the height of the monitor and their neck twist scores.

Both groups had a majority of participants that used a telephone for less than 30 min a day and were awarded a negative point, resulting in scores of one and two on this subscale. The scores for monitor and telephone were low because of the low telephone scores. As this section contributes to the final ROSA, a very low telephone score decreases the final ROSA score. However, a participant could experience a neck twist, a lengthy duration of time and the screen at a high level, thus scoring three. The low telephone score of one or two would result in a section score of two. This is a low-risk score. However, considering all of the literature that non-neutral and awkward postures are known risk factors for MSD (Devereux et al. 2002; Wahlstedt et al. 2010), and should be addressed, such a low score is misleading in a risk assessment. A minimum score of two points would be possible in the keyboard category. One point would be provided for neutral wrists, and as all participants would also be awarded a point for more than 4 h a day in front of a VDU, the second point would be awarded by this time factor. Scores increased with participants showing wrist extension, wrist deviation and with elevated shoulders. Elevated shoulders would be penalised twice, once at this section of the assessment, and again at the back-support section. Owing to the nature of the participants’ job requirements, none of the participants received a score for reaching up to overhead items.

The use of laptops generally influences this score on account of the added wrist extension, deviation and elevated shoulder in order to compensate for neck flexion. The same argument as for the monitor and telephone score applies to this section. As previously mentioned, even a low-physical risk factor should be addressed as a compounding effect emanating from multiple risk factors that affect MSD.

All participants used a pincer grip with the mouse. Duration scores varied, as not all participants required the mouse for the job tasks. Reach and palm rest also influenced these scores. The monitor and peripherals’ scores were derived from the keyboard and mouse score, as well as from the monitor and the telephone scores. Any score of five or more in this section would immediately result in a final ROSA of five or more.

The monitor and telephone scores were all under four for both groups. However, the effects of the keyboard score resulted in a higher score at this point. Even though the risk assessment programme does not indicate a high risk for MSD, it does not exclude the fact that participants may still experience risk factors that are not observed in the ergonomic risk assessment.

Using the ROSA as an assessment tool is not an independent measure of physical risk factors, but rather an overview of general workstation risk. Matos and Arezes (2015) noted that the scores in their study may be a result of not poor ergonomic equipment, but rather poor optimisation of the equipment. Sonne et al. (2012) found a correlation value or *r* = 0.36, a 13% rise in discomfort with a rising ROSA score, making the use of the ROSA to measure ergonomic workstation risk and MSD effective in relating MSD to ergonomic risk. The shortcomings of the ROSA do not diminish the value of the risk assessment for physical risks in ergonomic workstation set-up. When comparing the ergonomic risk factors of an MSD group to those of a reference group, the results of our study add to the body of knowledge that workstation set-up alone is not related to MSD (Coelho et al. 2015; Klussmann et al. 2008; Lima & Coelho 2018; Wu et al. 2012).

Physiotherapists and other healthcare professionals should use their clinical judgement when issuing ergonomic guidelines. The combination of physical and psychosocial risk factors increases the odds for MSD (Korhonen et al. 2003; Widanarko et al. 2014), and by acknowledging this, physiotherapists and other healthcare professionals should be cognisant of giving advice relating to ergonomics only on the basis of physical-risk factors. Assessment and interventions of work-related psychosocial factors should also be considered in the management of MSD.

## Strength and limitations

Validated outcome measures were used throughout this study. The ROSA was completed by the first author only, thus allowing for consistency in the assessments. Although not all of the data were collected on 1 day, the period of data collection was minimised as far as logistically possible. A sample of convenience was used which might not be a true representation of all VDU users in South Africa. The variety of professions and occupations within the various companies’ employees of the two sample groups did limit the generalisability of the findings. On account of the ROSA limitations and specificity, as well as the efforts of the participants to improve the workstation as the assessment was being conducted, the ergonomic risk assessment may have limited reach.

## Clinical implications

When educating patients using VDU in terms of their ergonomic workstation, the assessment needs to include all other relevant work-related risk factors, including but not limited to, job demands, job control, stress (psychosocial factors) and individual factors. As multi-site pain is common in individuals living with chronic pain, it could be hypothesised that as chronic pain is complex and multifactorial, interventions to address chronic pain may be warranted in MSD patients working with VDU.

## Conclusion

The use of ergonomic education for patients with MSD should be performed holistically, taking all the workplace-risk factors for MSD into account. As over-commitment is an indication of intrinsic factors and personal characteristics, the significant difference between the MSD group’s over-commitment score and that of the reference group suggests that interventions and methods to empower individuals are needed.
